# High-Throughput First-Principles Prediction of Interfacial Adhesion Energies in Metal-on-Metal Contacts

**DOI:** 10.1021/acsami.3c00662

**Published:** 2023-04-04

**Authors:** Paolo Restuccia, Gabriele Losi, Omar Chehaimi, Margherita Marsili, M. Clelia Righi

**Affiliations:** Department of Physics and Astronomy, University of Bologna, 40127 Bologna, Italy

**Keywords:** adhesion, high throughput, density functional theory, metal−metal interfaces, machine learning

## Abstract

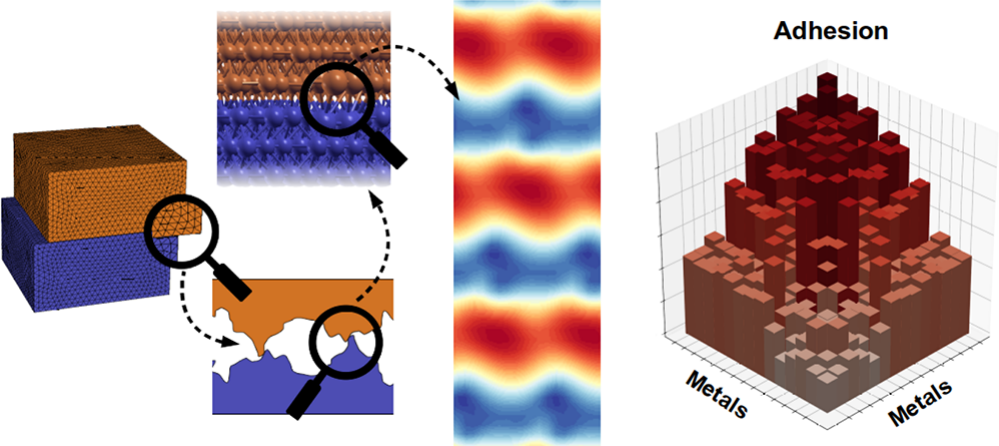

Adhesion energy, a measure of the strength by which two surfaces bind together, ultimately dictates the mechanical behavior and failure of interfaces. As natural and artificial solid interfaces are ubiquitous, adhesion energy represents a key quantity in a variety of fields ranging from geology to nanotechnology. Because of intrinsic difficulties in the simulation of systems where two different lattices are matched, and despite their importance, no systematic, accurate first-principles determination of heterostructure adhesion energy is available. We have developed robust, automatic high-throughput workflow able to fill this gap by systematically searching for the optimal interface geometry and accurately determining adhesion energies. We apply it here for the first time to perform the screening of around a hundred metallic heterostructures relevant for technological applications. This allows us to populate a database of accurate values, which can be used as input parameters for macroscopic models. Moreover, it allows us to benchmark commonly used, empirical relations that link adhesion energies to the surface energies of its constituent and to improve their predictivity employing only quantities that are easily measurable or computable.

## 1 Introduction

Adhesion energy, i.e., the energy gained when two surfaces make contact, is a key parameter affecting the mechanical behavior and failure of composite systems. The strength (or weakness) of an adhesive contact has a huge impact at a whole range of length scales: from geological natural faults where the adhesion energy between surfaces is modified by the presence of salt solutions affecting the frictional strength of the fault^1^ to tribolectric nanogenerators, novel devices able to convert otherwise lost mechanical energy into electricity: in this case the enhancements of surface charge generation have been linked to increased adhesion.^2^ Adhesive interactions are, in fact, particularly relevant in nanoscale systems, where the surface-to-volume ratio is high. For example, the functionality of nano- and micro-electromechanical systems (MEMS) is severely undermined by stiction. But indeed controlling adhesion is important in all phenomena and industrial processes in which the mechanical strength of an interface plays a role, for instance, in coatings where adhesion should be strong enough to bind the coating to the substrate throughout the component lifetime, in metallurgy where solid solubility in binary alloys, the so-called “metallurgical compatibility”, is strictly related adhesion,^3^ or, finally, in large-scale production of 2D materials where adhesion should be low enough to allow the mechanical exfoliation of monolayers from substrates.^4^ Adhesion, of course, plays a major role in tribology, the science of sliding surfaces: when a Lennard-Jones potential is used to describe the attraction force and the parameters of the model are constant, friction is found to be directly proportional to adhesion.^5^ In real systems this is not typically the case, and at the nanoscale, the shear strength is found to depend on the adhesion energy as a power law.^6^

Of course, the energy gained by forming a contact between two surfaces is influenced by macroscopic features such as the system geometry and surface roughness that determine the effective contact area. However, within a given contact area, the main driving forces that determine the strength of adhesion originate from the specific chemistry of the interface, namely the reactivity and willingness in bonding of the nanoasperity contacts. Indeed, adhesion energy is directly controlled by the atomistic structure of the interface and, as such, can be influenced by microscopic details such as crystal orientation and stacking, presence of adsorbates, impurities, defects, and segregates.^7−11^ Hypothetically, the knowledge and control of these nanoscale contacts could allow the understanding, anticipation, and optimization of the mechanical behavior of interfaces. However, such knowledge and control are extremely difficult to gain from an experimental point of view. This is the reason why, like in many other fields where the properties of interfaces play an important role, such as catalysis,^12^ energy storage,^13^ corrosion,^14^ microelectromechanical systems,^15^ etc., also in this case combining theoretical calculations and experimental evidence is crucial.^16−19^

Moreover in recent times, alongside the fundamental combination of theory and experiments on specific problems, high-throughput density functional theory (DFT) approaches are used in materials design and discovery.^20−25^ Increasingly complex DFT-based high-throughput workflows have been developed, screening molecular adsorption energies and sites on intermetallic surfaces in view of electrochemical catalysis applications,^26^ looking for novel 2D superconductors,^27^ predicting lattice parameters and formation energies of high-entropy alloys,^28^ and identifying promising metal–organic frameworks for heterogeneous catalysis.^23,29^ However, in this quickly developing framework, a systematic study of mechanical and tribological properties of solid–solid heterointerfaces has not been addressed yet. Most probably this is due to the inherent difficulties that this kind of system poses and to the fact that the community of references (the tribology, metallurgy, and mechanical manufacturing communities) most of the time relies on classical macroscopic engineering models.

**Figure 1:**
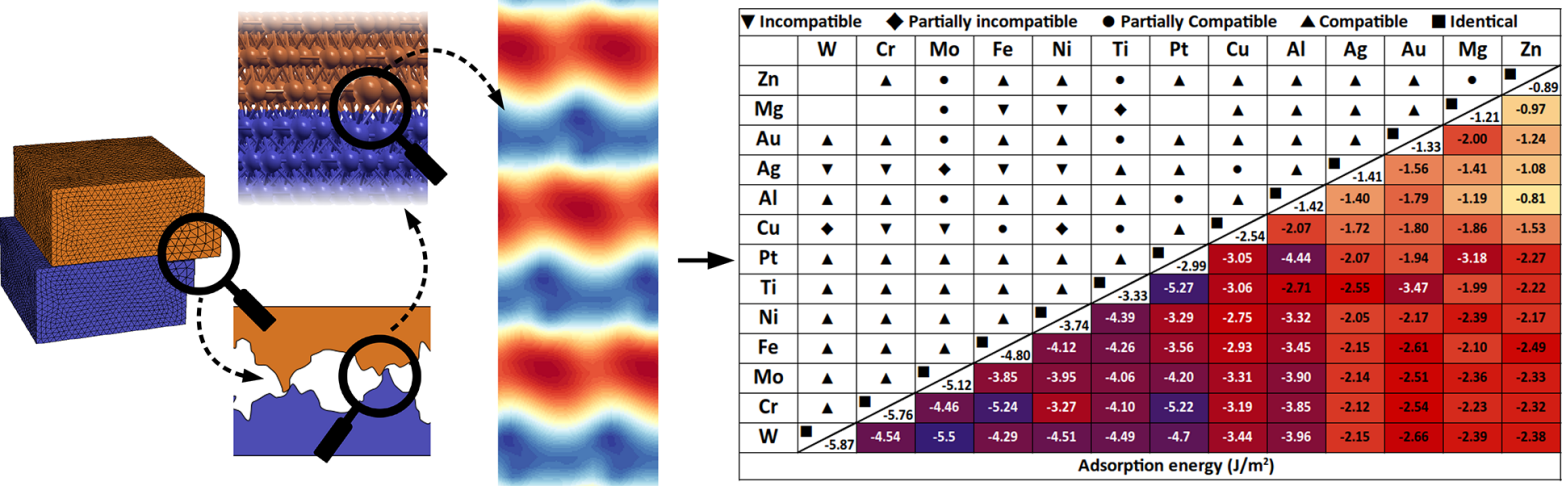
Overview of the present work: nanoasperities, present in any mechanical contact, are simulated atomistically as two interfaced slabs (left panels). The electronic properties of the interface define a 2-dimensional potential energy surface (PES) that regulates the lateral shifts of the surfaces (central panel). From PES minima, *ab initio* predictions of adhesion energies can be obtained turning circles and triangles into accurate numbers (right panel). The left and central panel refer to the Cu/Fe interface.

Indeed, despite the importance of metal/metal heterointerfaces in a variety of applications (automotive, catalysis,^30^ nuclear fusion generators,^31−33^ magnetic nanodevices^34,35^), up to now researchers and engineers attempting to use a more systematic and analytical approach can only rely on empirical and qualitative data, employing the concept of “metallurgical” or “tribological compatibility” to estimate the work of adhesion between different metals and on a qualitative table, similar to the one presented in Figure 1, containing filled, half-filled, or empty circles to describe such behavior.^3,36,37^

**Figure 2:**
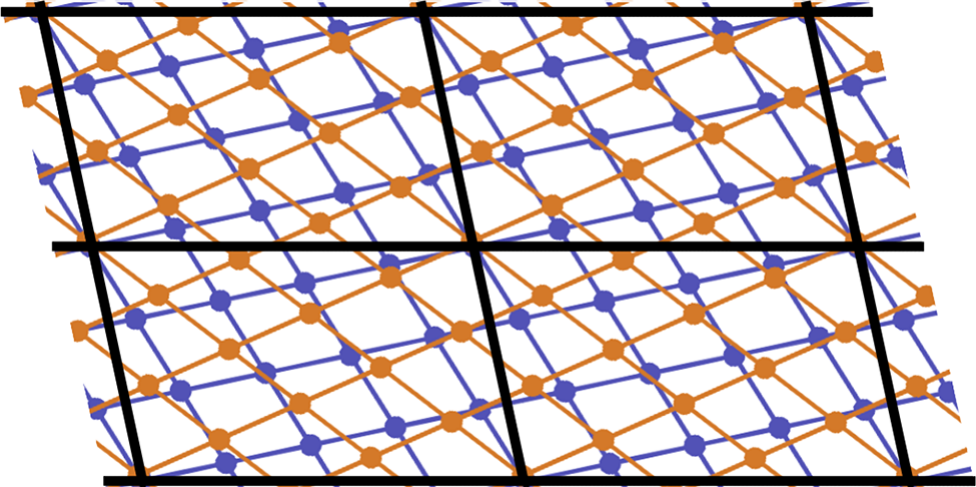
Top view of the interfacing atomic layers of the Fe/Cu interface. The blue (orange) points and lines represent the Fe (Cu) atoms and unit cells, respectively. The solid black lines are the boundaries of the supercells.

In the field of tribology, the only systematic DFT analysis performed so far just considered the interfacial adhesion energy of homogeneous interfaces.^38−40^ The *ab initio* simulation of heterogeneous interfaces is indeed especially challenging from an high-throughput perspective, both for the design of an effective workflow and for the higher computational cost that such simulations demand. Because solid-state *ab initio* codes implement periodic boundary conditions, the simulation of a heterogeneous interface requires matching, within the same supercell, two different lattices. Therefore, to limit the lattices strain and/or stress within reasonable values, large supercells (of up to hundreds of atoms) are used, increasing the computational cost of each simulation (see Figure 2). Moreover, the calculation of adhesion energies requires, for each interface, the identification of the energetically most stable configuration. This is obtained by sampling the possible lateral displacement between the two faces and further increases the computational load by at least an order of magnitude.

As pictorially shown in Figure 1, here we present the first application of a novel high-throughput *ab initio* workflow specifically developed by our group to systematically search for optimal solid–solid interface geometries and accurately determine interfacial adhesion energies. We use it for the determination of the adhesion energy, *E*_adh_, of around a hundred metallic heterostructures, ranging from transition to noble metals, turning the filled and empty circles of the compatibility tables into accurate numbers. To perform this task, we developed TribChem,^41^ a modular scientific code relying on Fireworks^42^ and connected to publicly available databases, to automatically generate the structures and run the density functional theory (DFT) simulations.

The data set of adhesion energies allowed us to identify general trends of *E*_adh_, where the atomic species with partially occupied d-orbitals show a higher adhesion. Our data set confirms that adhesion energies can be reasonably well inferred from the knowledge of the surface energies of the two interface constituents. To obtain a more predictive expression, we used a machine learning approach and combined the *E*_adh_ data set with the databases of single-component properties such as the bulk modulus, surface energy, and bulk cohesive energy stemming from much less expensive calculations. This allowed the determination of a predictive formula for *E*_adh_ as a function of intrinsic features of the heterostructure constituents alone, which can prove useful for the preliminary estimation of the *ab initio* adhesion energy of new heterostructures avoiding expensive supercell calculations.

## 2 Methods

The high-throughput screening of the adhesion of solid heterointerfaces has been performed using the TribChem software developed by our group; complete details of the code are presented in a separate work.^41^ TribChem automatically creates input files and geometries, submits and runs simulations, collects data in external databases, and analyzes them. It is a Python-based code employing extensively Fireworks^42^ and Atomate^43^ as workflow managers. The manipulation of the input as well as pre- and postprocessing operations is performed with the help of the Pymatgen^44^ and MPInterfaces.^45^

DFT calculations are run by TribChem using the Vienna Ab initio Simulation Package (VASP) .^46−49^ Being a widely used, universal functional with a reasonable accuracy,^50^ the Perdew–Burke–Ernzerhof generalized-gradient approximation (PBE-GGA) of the exchange-correlation (xc) functional^51^ has been employed, and ultrasoft pseudopotentials from the VASP suite have been used.^52^ Of course, our interfacial adhesion energy database can be expanded to systematically look at the effects of different approximations on the xc functional and of the introduction of dispersion forces, thus contributing to the ongoing debate on the best functional for the description of surface and interface properties.^50,53−56^ However, this is beyond the scope of the present work and will be the subject of further investigation.

### 2.1 Workflow Unit

In this study, we considered elemental crystalline bulks to extend our previous work of homogeneous interfaces to heterogeneous solid–solid contacts. We created these heterogeneous structures by matching two surfaces that differ in both composition and orientation. We considered the most relevant elements for industrial applications, such as transition metals, and analyzed their structural and tribological properties. In particular, the workflow generated many figures of merit, namely the bulk cohesive energy, bulk modulus, surface energy, adhesion energy, and charge transfer upon interface formation, which are relevant for tribological studies.

**Figure 3:**
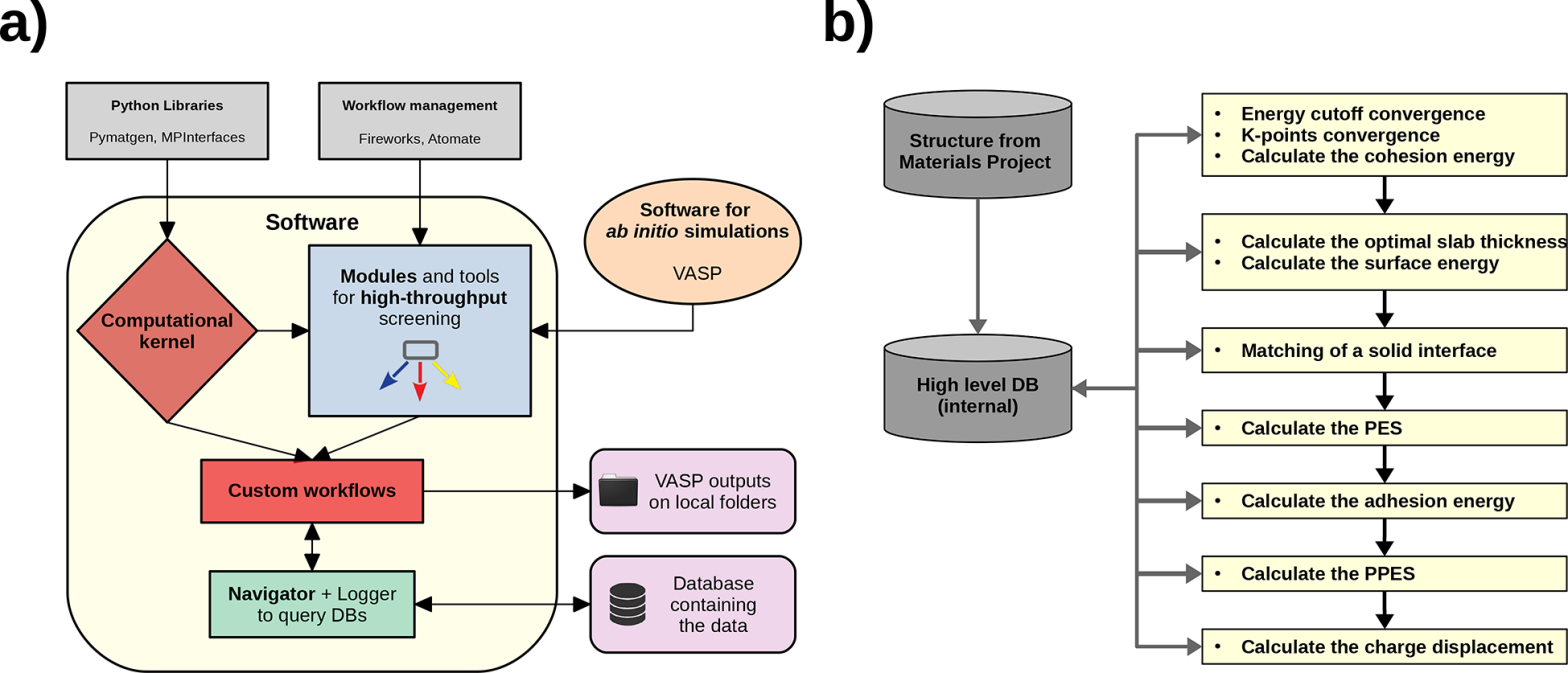
Structure of our software showing the different modules composing the code (a) and flow diagram explaining the sequence of logical steps followed by our computational workflow to calculate the properties of solid interfaces (b).

The workflow schematic is shown in Figure 3b. The process starts by retrieving the input structure for the materials of interest from online repositories (in particular, we used the information provided by the Materials Project database^57^) and storing them in our database.

The elemental bulks are optimized by converging their lattice parameters via a fit with the Birch–Murnaghan equation of state^58^ and optimizing the kinetic energy cutoff and *k*-point density mesh required by DFT. Then the slabs are obtained by cutting the bulk along a specific direction. In our case, we selected the most stable orientation for the different crystalline structures, namely the (111) for FCC crystals, the (110) for BCC, and the (0001) for HPC. The optimal thickness for the slabs is calculated by changing the number of layers and checking when their surface energy converges.

As better detailed in the next subsection, a modified version of the MPInterfaces library^45^ is used to match the two slabs and form the heterogeneous interfaces. An example of such matching is shown in Figure 2.

Once the interface is generated, the interaction energy is computed for different relative lateral positions to find the interfacial adhesion energy, which corresponds to the energy minimum. The lateral shifts are identified as those that pair the high symmetry points of the two mating surfaces. Depending on the interface, either homogeneous or heterogeneous, the number of nonequivalent relative lateral positions is 6 or 16, respectively. The interaction energy is computed as
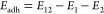
1where *E*_12_ is the energy of the heterogeneous structure, *E*_1_ is the energy of the lower slab, and *E*_2_ is the energy of the upper slab. The final adhesion energy is obtained from a fully relaxed calculation of the interface and the of two isolated surfaces.

### 2.2 Surface Matching

The surface matching, performed by the MPInterfaces library,^45^ is based on the Zur algorithm,^59^ which builds the lowest area supercell meeting a series of criteria (on the lattice sides and angles strains and on the maximum allowed areas).

As reported in the original paper, starting from the consideration that the unknown supercell that matches the two original lattices (labeled 1 and 2 for clarity) has an area *A* that is an integer multiple of the respective *A*_1_ and *A*_2_ areas, and that thus ideally *A* ≈ *n*_1_*A*_1_ ≈ *n*_2_*A*_2_, so that *n*_1_/*n*_2_ ≈ *A*_2_/*A*_1_, the algorithm reasoning follows these steps:

- All the sets of integers (*n*_1_, *n*_2_) that satisfy the following conditions are identified:
  (i) The ratio *n*_1_/*n*_2_ approximates *A*_2_/*A*_1_ within a given tolerance (in our case 5%).
  (ii) The area *A* ≈ *n*_1_*A*_1_ ≈ *n*_2_*A*_2_ is less than a maximum allowed value (in our case 190 Å^2^).
- For each couple (*n*_1_, *n*_2_) all possible superlattices of the lattice 1 and area *n*_1_*A*_1_ and, similarly, all possible superlattices of the lattice 2 and area *n*_2_*A*_2_ are built.
- Because different sets of primitive translations can be used to generate the same lattice, a reduction scheme is applied to all the previously found superlattices. In this way a specific set of primitive translations is selected in a unique way among all the possible equivalent sets for each superlattice.
- The reduced superlattices stemming from lattice 1 are compared to those stemming from lattice 2 to find the ones with a mismatch that is at most 2% for the sides and 0.01° for the angles.
- Among the “matching” superlattices, the one with the lowest area is selected.

The applied strain is inversely proportional to the bulk modulus of the materials. In this way, we allow the materials with a higher compressibility to have larger deformation during the interface creation.

### 2.3 Database Structure

The database is the central element of our workflow. In particular, we used MongoDB—a NoSQL database based on a key-value document structure to store and retrieve data. A significant data flux flows from and to the database during any calculation. It is then crucial to develop an efficient structure to retrieve the data during the workflow execution and perform the subsequent analysis of the results. To make the management more efficient, we created a new (high-level) database where we only saved the physical results. In this way, the workflow execution, i.e., the internal database of the Fireworks package, is separated from the storage of the physical results. Separating the two databases improves the results analysis and their sharing with the scientific community.

## 3 Results and Discussion

Taking into the account a subset of technologically relevant transition metals, to which simple metals Al and Mg were added due to their relevance in alloy,^60^ the most stable faces of Ag, Al, Au, Cr, Cu, Fe, Ir, Mg, Mo, Ni, Pt, Rh, Ti, V, W, and Zn have been mated to form 16 homogeneous and 120 heterogeneous interfaces. Following the assessment of our computational setup over bulk and homogeneous interface properties (as shown in the Supporting Information), the heterogeneous interfaces were obtained making use of the matching algorithm proposed by Zur,^45,59^ as explained in the Methods section. An example of the matching is shown in Figure 2, for the Cu/Fe interface, where the superlattice and substrates unit cells are shown.

Once the optimal supercell is found, the top and bottom slabs can be shifted by different amounts one with respect to the other. By exploring the different shifts for which the high-symmetry points of the two surface match and collecting the corresponding total energies, a 2-dimensional potential energy surface (PES) is built (see central panel of Figure 1).^40^ The identification of the minima in the PES allows the calculation of the adhesion energy, being , where *A* is the supercell area, *E*_12_^min^ is the total energy of the optimized structure of the two slabs in contact, and *E*^1^ (*E*^2^) is the total energy of the isolated upper (lower) slab. Is worth noting that the calculated PES allows the computation of important tribological figures of merit such as the ideal shear strength and stacking fault energy.^61,62^ In the central panel of Figure 1, the PES of the Fe/Cu interface is shown. Because of the reduced degree of commensurability, the PES corrugation, i.e., the energy difference between maxima and the minima, of heterogeneous interfaces is typically 1 order of magnitude less than in the cases of homogeneous interfaces PES.^40^

### 3.1 Adhesion Database

**Figure 4:**
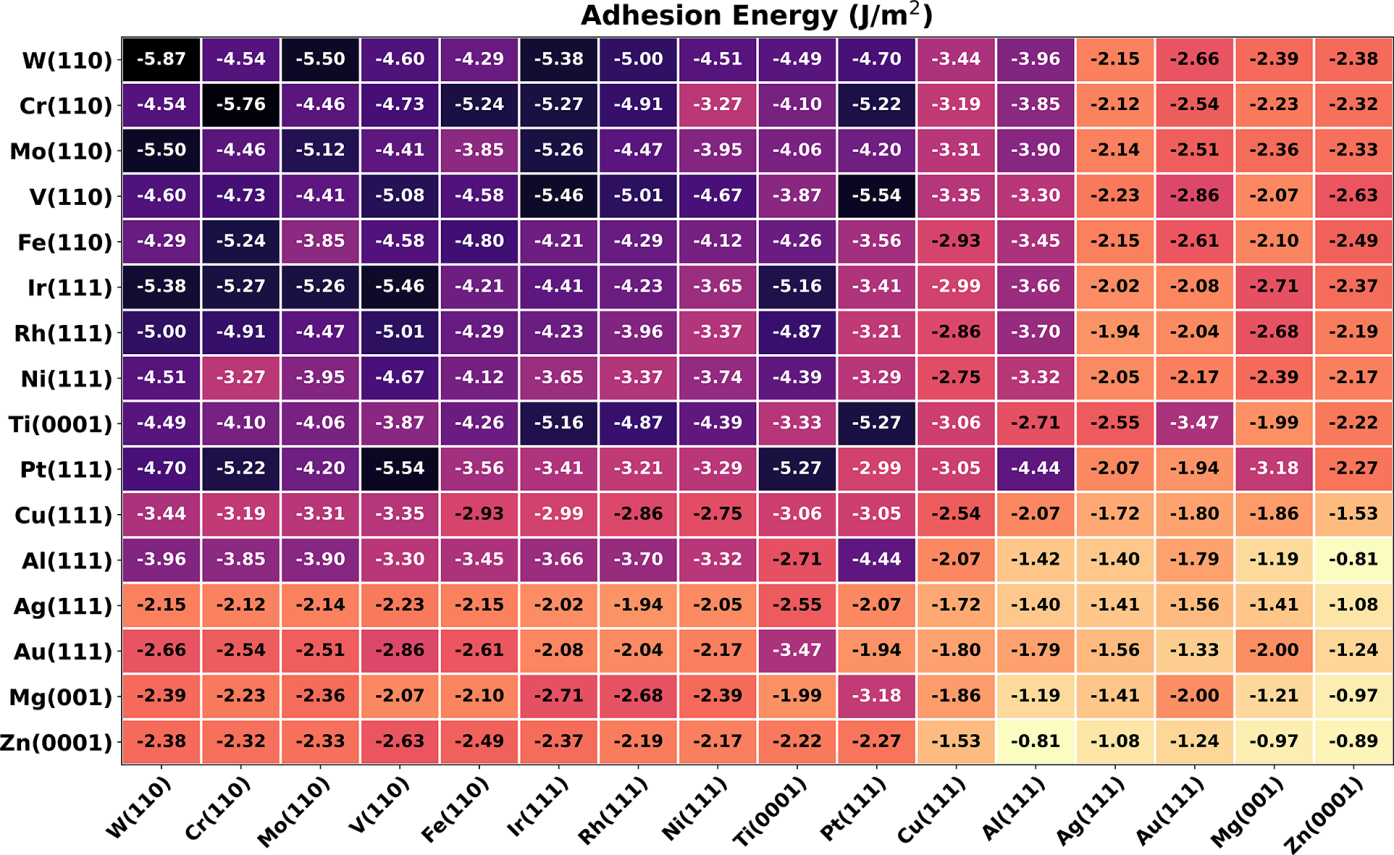
Adhesion energies data set for the heterogeneous interfaces. The data are ordered following the value of *E*_adh_ of the corresponding homogeneous interfaces. We find W and Cr to the left, presenting the higher *E*_adh_, which decreases moving rightward toward Mg and Zn. Darker colors are related to higher *E*_adh_.

The adhesion energies of the 120 interfaces are listed in Figure 4. In the table, the elements are ordered with respect to the strength of adhesion of the corresponding homogeneous interfaces, the color scheme marks with darker color interfaces with higher adhesion.

The resulting *E*_adh_ matrix is clearly divided into blocks where the *E*_adh_ values of the heterostructures are strictly related to those of the homogeneous cases: the higher the homogeneous adhesion for the two components, the higher the resulting heterogeneous adhesion after the matching. In the top left corner, we find materials with incomplete d-shell that show a larger adhesion. The bottom right corner presents a combination of materials with fully occupied d-orbitals, like noble metals, with lower adhesion energies. This behavior is consistent with the so-called d-band center theory for molecular adsorption on metals, which explains the lower reactivity of noble metals.^63,64^

In general, high-throughput approaches can bring to light correlations between different properties of the studied systems. In our case of particular interest are the correlations between ground-state electronic properties of the interface and *E*_adh_. In previous studies for homogeneous structures^6,65^ we have shown a direct correlation between *E*_adh_ and the charge transfer upon interface formation (ρ_disp_), given by the following expression:
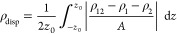
2where 2*z*_0_ is the interface distance, ρ_12_ is the planar average of the interface charge density, and ρ_1_ (ρ_2_) is the planar average of the charge density of the bottom (top) slab of the specific system. ρ_disp_ quantifies the accumulation of charge at the interface.

**Figure 5:**
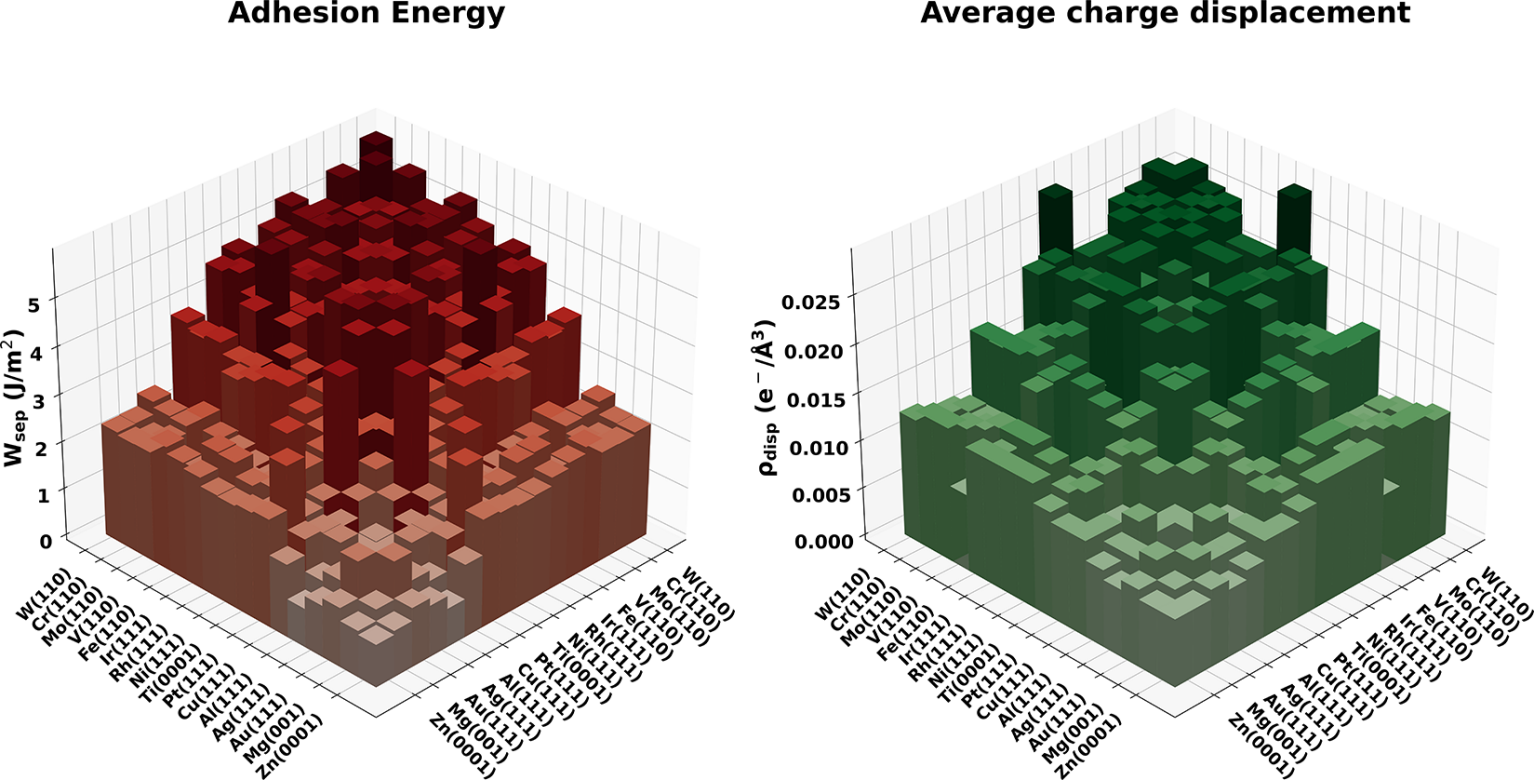
Bar plot of *E*_adh_ (left panel) and ρ_disp_ (right panel) for the different heterostructures. The data ordering is the same as Figure 4; the color scales are related to the magnitude of *E*_adh_ and ρ_disp_.

By comparing charge transfer values with the adhesion energies, shown as histograms in the right and left panels of Figure 5, is clear that also for heterostructures an important correlation between the two quantities is present. More quantitatively, by performing a linear regression of *E*_adh_ against ρ_disp_, a correlation coefficient *R*^2^ of 75% is found.

### 3.2 Adhesion Energies from Intrinsic Constituent Properties

To avoid expensive simulations of the entire interface, an empirical relation connecting *E*_adh_ to intrinsic properties of the single components would prove extremely useful. Indeed, within simple physical models,^66^ adhesion energy is linked to the surface energies of the two constituents, γ_1_ and γ_2_, via the interfacial energy γ_12_, namely *E*_adh_ = γ_12_ – γ_1_ – γ_2_.^67,68^ Physically γ_12_ is the energy required to generate an unit of area of interface between materials 1 and 2 starting from the corresponding bulks.

Following ref (67) and using a combining rule approximation for the interfacial energy , we obtain that the adhesion energy is given by the geometric mean (GM) of the surface energies of the single components, namely . Testing this relation against our data set, we find that it fits reasonably well our results with a correlation coefficient *R*^2^ = 0.80 and a root-mean-square error (RMSE) of 0.57 J/m^2^, as shown in the Supporting Information.

In order to extend the simple analytical formula *E*_adh_ = −2γ_GM_ and identify suitable descriptors that can improve the predictivity of such a model, machine learning algorithms can be applied to our data set. Indeed, in recent years, many works on adsorption energy prediction based on machine learning approaches have been presented.^69−73^ One of the most advanced technique in predicting materials properties is the so-called Sure Independence Screening and Sparsifying Operator (SISSO) algorithm,^74^ which can identify the best descriptors and the optimal mathematical expression fitting the training data. The SISSO algorithm works on features spaces (called **Φ**_***n***_) containing the identified descriptors and mathematical operations, and it combines them to generate the optimal fitting expression and can generate effective models with limited training data sets.^75−77^

We thus used the SISSO algorithm to obtain simple mathematical expressions for predicting *E*_adh_ in heterointerfaces using only properties of the single components. Although surely increasing the predictive capacity of the model, quantities such as charge displacement, which are properties of the whole interface, were disregarded because their determination would require the same computational effort of the *ab initio* calculation of the adhesion energy itself. In particular, as possible descriptors, we screened the two materials cohesive energy ϵ_1,2_, bulk modulus *K*_1,2_, surface energy γ_1,2_, atomic density at surface ρ_1,2_^*a*^, in plane surface area *A*, the arithmetic (AM) and geometric averages (GM) of these descriptors, and the electronegativity difference (χ_1_ – χ_2_). The algorithm search was also limited, in terms of mathematical operators, to algebraic functions, somewhat limiting the algorithm capability but, at the same time, increasing the readability and generality of the expressions. Namely, we obtained the simple linear relationship, **Φ**_**0**_, and two nonlinear formulas, **Φ**_**1**_ and **Φ**_**2**_, given by
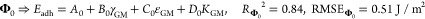
3
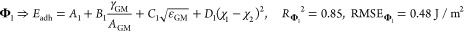
4
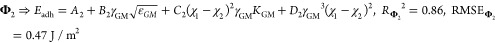
5The SISSO algorithm identified γ_GM_, ε_GM_, and *K*_GM_ as the best predictors in a simple linear relationship **Φ**_**0**_ and in the nonlinear formula **Φ**_**0**_. In **Φ**_**1**_ also the electronegativity difference plays a role.

**Figure 6:**
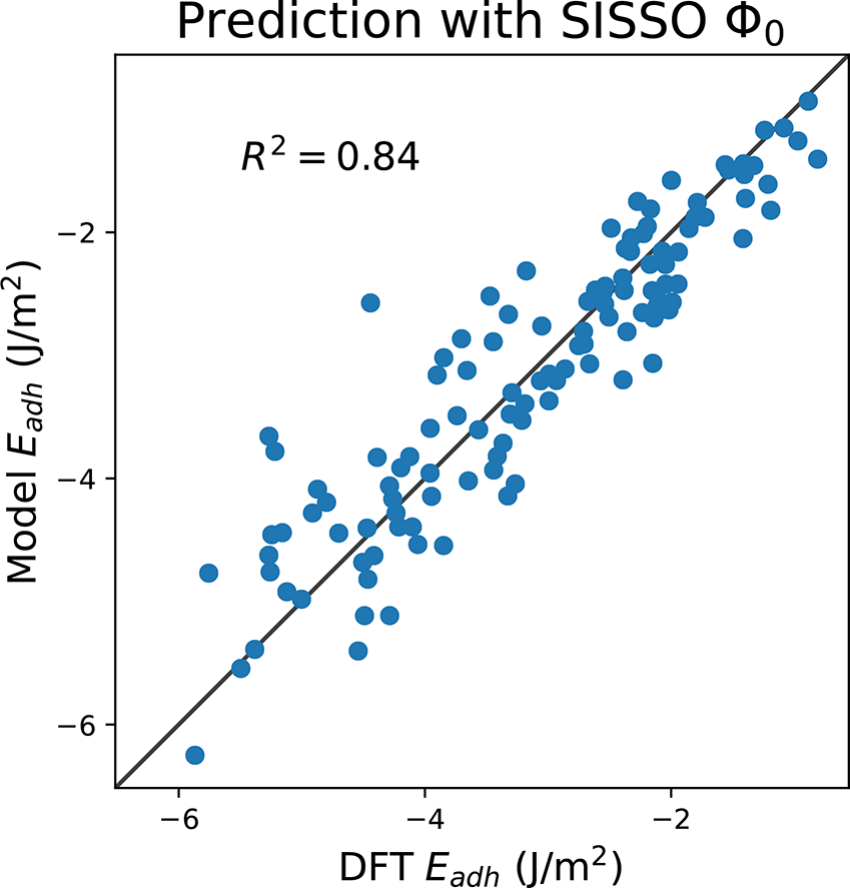
Parity plot of the SISSO **Φ**_**0**_ data model against the DFT *E*_adh_ calculations. The black solid line represents the parity between the DFT data and the predicted value. As an inset it is also reported the correlation coefficient and the numerical values of the regression coefficients.

The table reporting the coefficients *A*_*i*_, *B*_*i*_, *C*_*i*_, and *D*_*i*_, determined by the SISSO algorithm, can be found in the Supporting Information; the coefficients of **Φ**_**0**_ are also reported in Figure 6. The reported values are obtained using the entire set of interfaces as training set; the related learning curves are shown in the Supporting Information. The correlation coefficients *R*^2^ and the root-mean-square error (RMSE) associated with each expression, listed below each equation, obviously show how the correlation between predicted and computed data increases upon increasing the complexity of the formula at the price of physical interpretation. Instead, considering (**Φ**_**0**_), a simple linear relation between *E*_adh_ and the three descriptors ϵ_GM_, *K*_GM_, and γ_GM_, the physical content is clear: the work of adhesion (−*E*_adh_) tends to be larger when both surfaces have high surface energy and high bulk cohesive energies (the geometric mean is multiplicative) whereas stiffness gives a negative contribution. Indeed starting from specific bulk bonding strengths, taken into account by ϵ_GM_, the geometry (i.e., the orientation) of the facing surfaces plays a role through γ_GM_, and finally harder materials are less inclined to deform in order to build a stronger interface. In Figure 6 the parity plot of eq 3 against the computed *E*_adh_ data set is shown; the correlation is significant and slightly higher than that found with a simple linear regression of *E*_adh_ against γ_GM_. Of course, different strategies could be adopted to reduce the RMSE and increase the SISSO models predictivity: (i) SISSO could be allowed to use more and more complex expressions; as we already mentioned, we decided to avoid this strategy as we found the proposed models less and less apt to a physical interpretation the more complexity of expression was allowed. (ii) Materials could be grouped in more homogeneous sets. For instance, as reported in the Supporting Information, we tested the SISSO algorithm against a set in which we removed Al, Mg, and Ti, the first being the only simple metals present in the original set and the latter because it required an exceedingly large number of atomic layers to get a converged surface energy influencing the calculation of adhesion performed on thinner slabs. Indeed, in this way we were able to reduce the RMSE by 24% using a simple linear expression and between 20% and 23% for more complex models. (iii) Different kinds of trial descriptors could be provided to the SISSO algorithm. As reported in the Supporting Information, we added information on the geometric commensurability between the two surfaces, namely the ratios between the areas of the original cells and that of the interface. In this way the RMSE was further reduced by around 3%. Another possible class of descriptors concerns the electronic properties of the two materials. Indeed, the search for other valuable descriptors is a very important topic in view of enhancing the predictivity of the model and will be the subject of further investigations being beyond the scope of the present article.

### 3.3 Conclusions

In summary, we used the high-throughput *ab initio* workflow implemented in our software TribChem to screen the adhesion energies of more than 100 metallic heterogeneous interfaces, creating an accurate database. This systematic approach is a significant improvement compared to previous ones, based on qualitative “compatibility” parameters between different materials.^37^ The metallic heterostructures were automatically generated by mating two surfaces, and their optimal relative lateral position was detected by creating a PES landscape, the absolute minima defining to the adhesion energy. The highest adhesion is provided by species with partially filled d-orbitals, whereas the lowest values are obtained with noble metals. The computed adhesion energies correlate well with the charge transfer, an interface property, and with the geometric average of the surface energies.

We applied one of the most advanced machine learning approaches, the SISSO algorithm, to the generated data set to identify a simple linear formula to predict the heterostructure adhesion in terms of intrinsic properties of the single interface component alone. This relation allows a preliminary estimation of adhesion energy without requiring to run expensive *ab initio* supercell simulations.

This study focused on clean, perfectly flat metallic heterointerfaces at zero temperature. Evaluating the accuracy of the approach by direct comparison with experiments is a rather complex task as pointed out in ref (39). Difficulties arise from the scarcity of experimental results and for the realistic experimental conditions. To better contextualize the accuracy of our approach then, it is worth taking a step backward and consider its relation to calculations of surface energy (a quantity which is more frequently measured, in more controlled setups). The surface energy can be obtained as half adhesion energy of homogeneous interfaces; this is true for our calculated values within a mean absolute error of 0.15 J/m^2^. On the other hand, our surface energies compare well with the theoretical values published in the Material Project Database,^57^ with a mean absolute error of 0.03 J/m^2^. In turn, the Material Project database results are in excellent agreement with the extrapolated experimental values, with an average underestimation of only 0.01 J/m^2^.^78^ In this perspective, this approach for the calculation of the ideal intrinsic adhesion can be considered accurate as much as DFT calculations are accurate in the determination of surface energies.

Exposure to air or other experimental and operating conditions may easily promote the formation of an oxide layer on the metal surfaces, drastically altering adhesion. While the adhesion energy between flat surfaces is essential to analyze and model nanoasperities^79,80^ and to create effective models to describe frictional hysteresis loops,^81^ in regards to the temperature and contamination issues this study provides upper limits to the adhesion energies. However, the workflow can straightforwardly be extended to include oxide layers, contaminants, adsorbates, and other classes of materials such as oxides and semiconductors. At the same time, starting from zero temperature data, it is possible to extrapolate tribological relevant quantities, such as the ideal shear strength, to finite temperature.^82^ In these perspectives this work can be seen as a first application of a novel DFT high-throughput workflow devoted to solid–solid interfaces, which will be relevant, in its extensions, to a variety of fields ranging from geology to nanotechnology.

## Supporting Information Available

Additional data on the accuracy of bulk and surface energy calculations; raw data for charge redistribution for each interface; linear regression using γ_GM_ against all data; SISSO coefficients; SISSO predictions with different data sets and descriptors (PDF)
