# Supplementary material for: High-Throughput First-Principles Prediction of Interfacial Adhesion Energies in Metal-on-Metal Contacts
Source: ACS Appl Mater Interfaces. 2023 Apr 4;15(15):19624–33. doi: 10.1021/acsami.3c00662 (PMC10119859; doi:10.1021/acsami.3c00662)
Supplement: Supplementary file 1 — am3c00662_si_001.pdf [file am3c00662_si_001.pdf]

Supporting Information: High throughput first principles prediction of interfacial adhesion energies in metal-on-metal contacts

Paolo Restuccia, Gabriele Losi, Omar Chehaimi, Margherita Marsili, and M. Clelia Righi*

Department of Physics and Astronomy, University of Bologna, 40127 Bologna, Italy

E-mail: clelia.righi@unibo.it

1 Bulk and surface properties

First, we checked the accuracy of the data generated by the workflow. We have already shown the capability of the previous version of the workflow,[S3] but we want to check that everything works properly with the structures of interest. To do this, we computed the bulk and surface properties of the homogeneous interfaces. The results are shown in Fig. S1. They report parity plots of the computed lattice parameter, cohesive energy, bulk modulus and surface energy of the homogeneous systems against the reference values. It is possible to notice that for all the considered quantities, we obtain an excellent agreement between the computer data and the reference values, with a correlation coefficient ranging from 0.90 to 0.99. This test proves the high reliability of our workflow in computing the physical bulk and surfaces properties.

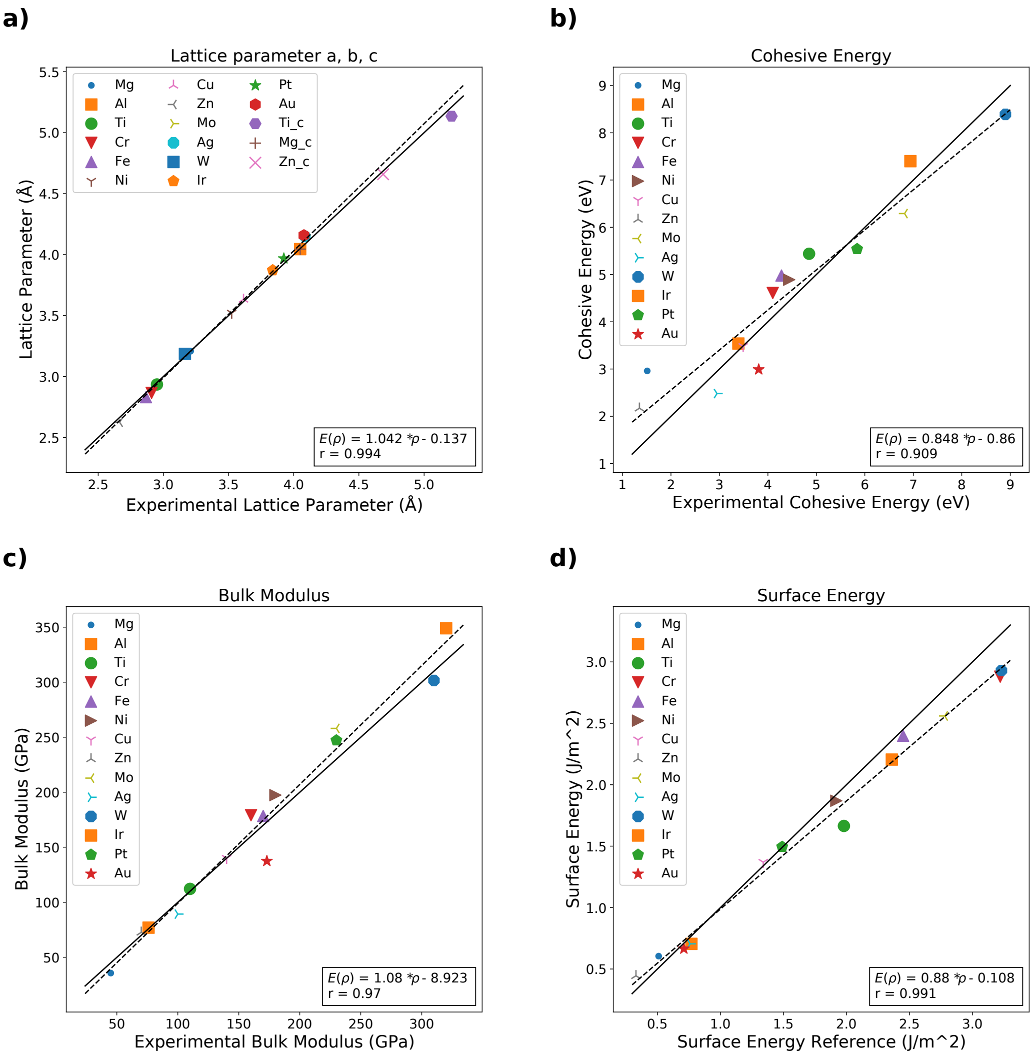

Figure S1: Comparison with the reference values for the lattice parameter, bulk modulus, cohesive energy, and surface energy. The reference values for the lattice parameter, the cohesive energy and bulk modulus are from Ref.,[S1] whereas the surface energies are taken from the Materials Project database.[S2]

2 Raw data for charge redistribution

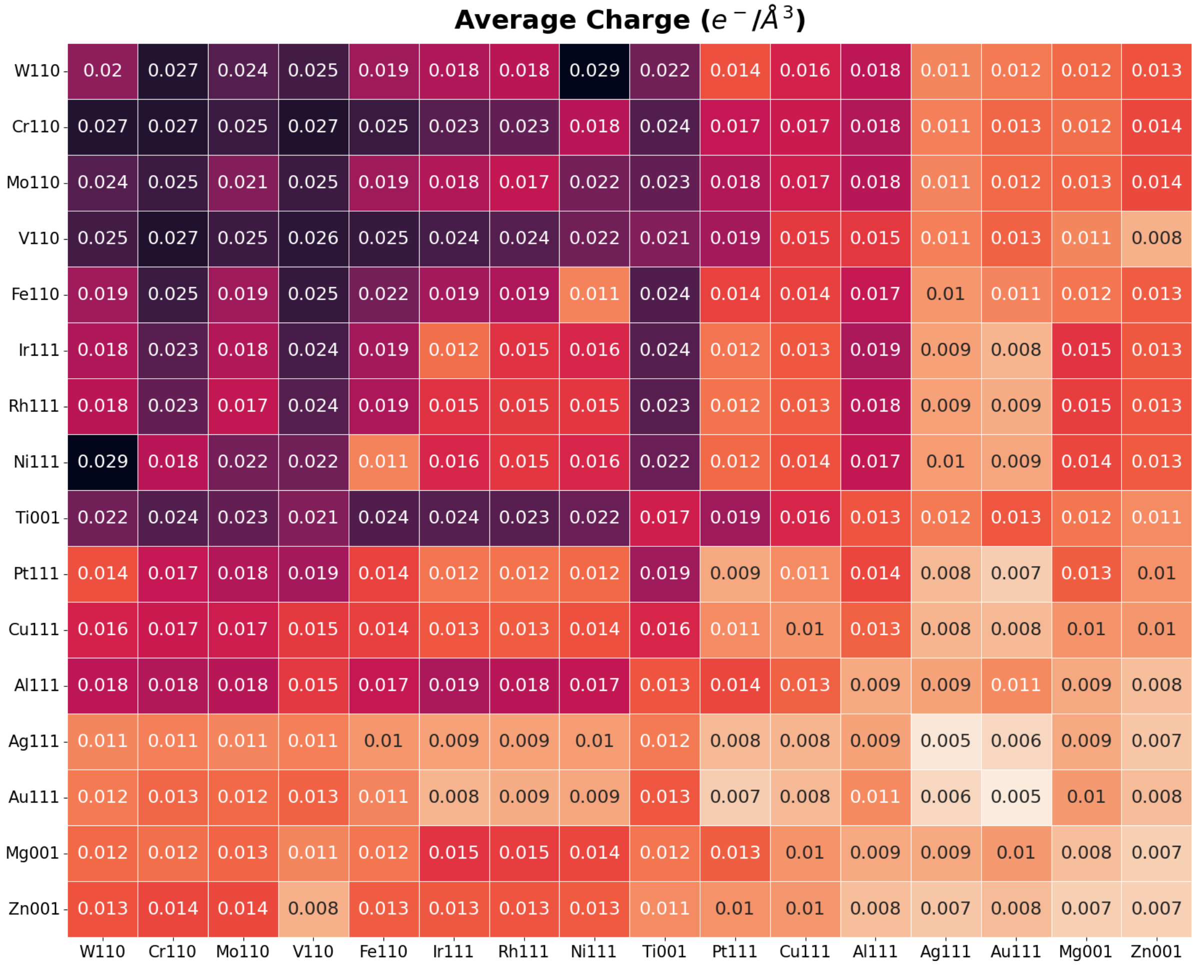

Figure S2: Raw data for charge redistribution calculated for the heterogeneous interfaces. The data are ordered following the value of E_{adh} of the correspondent homogeneous interfaces. Moving along the columns, the colour scale helps understanding how the charge transfer varies when the particular surface is matched to all the other ones.

3 Linear regression E_{adh} against γ_{GM}

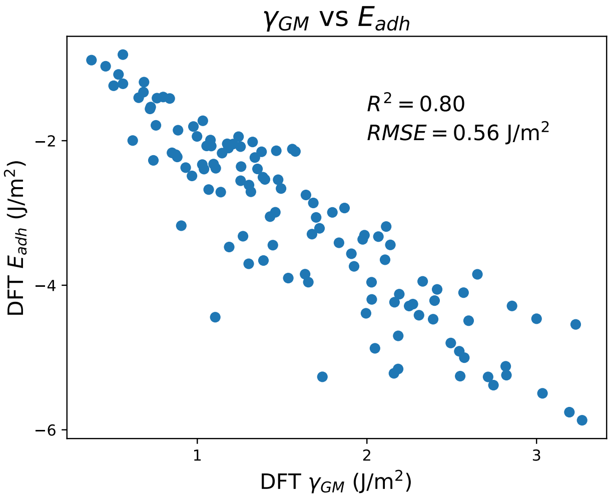

Figure S3: Parity plot of the computed E_{adh} against γ_{GM}. The black solid line represents the parity between E_{adh} and γ_{GM}. The inset shows the correlation coefficient and the root mean square error (RMSE).

4 Coefficients from the SISSO algorithm

We report here the correlation coefficients, the root mean square errors and the best fit coefficients for the formulas $\mathbf{\Phi_0}$, $\mathbf{\Phi_1}$, $\mathbf{\Phi_2}$ found by the SISSO alghoritm in order to express E_{adh} as algebraic functions of single components properties.

Table S2: Coefficients with corresponding units for Eq. 2, 3 and 4 of the main manuscript.

Model	R^2	RMSE (J/m^2)	MAE (J/m^2)
$\mathbf{\Phi}_0$	0.84	0.51	1.87
$\mathbf{\Phi}_1$	0.85	0.48	1.73
$\mathbf{\Phi}_2$	0.86	0.47	1.70

Model	A_i	B_i	C_i	D_i
$\mathbf{\Phi}_0$	$0.26\ \frac{\mathrm{J}}{\mathrm{m}^2}$	-1.07	$-0.46\ \frac{\mathrm{J}}{\mathrm{m}^2\ \mathrm{eV}}$	$0.0028\ \frac{\mathrm{J}}{\mathrm{m}^2\ \mathrm{GPa}}$
$\mathbf{\Phi}_1$	$2.43\ \frac{\mathrm{J}}{\mathrm{m}^2}$	$-5.87\ \mathrm{\AA}^2$	$-1.87\ \frac{\mathrm{J}}{\mathrm{m}^2\ \sqrt{\mathrm{eV}}}$	$-0.61\ \frac{\mathrm{J}}{\mathrm{m}^2}$
$\mathbf{\Phi}_2$	$6.90\ \frac{\mathrm{J}}{\mathrm{m}^2}$	$-0.63\ \frac{1}{\sqrt{\mathrm{eV}}}$	$-0.0026\ \frac{1}{\mathrm{GPa}}$	$0.19\ \frac{\mathrm{m}^4}{\mathrm{J}^2}$

5 Learning curves for SISSO generated formulas

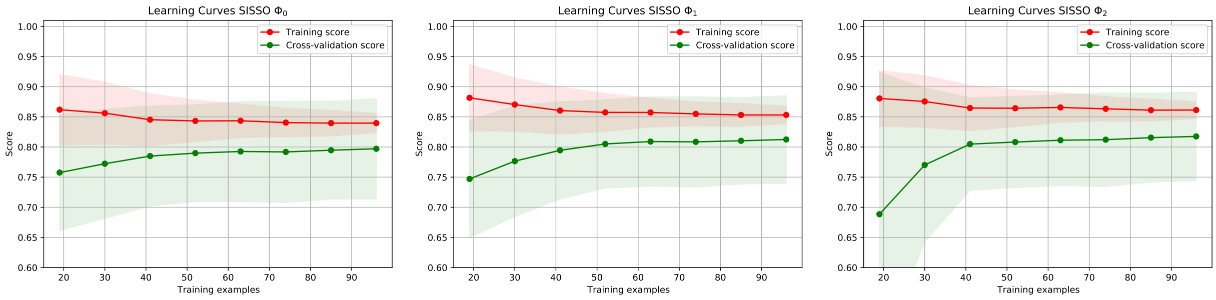

Figure S4: Learning curves for the three relationships identified with the SISSO algorithm, Φ_0 (left panel), Φ_1 (center panel), Φ_2 (right panel). The red (green) curves represent the training (validation) score for each model.

We can test the effectiveness of the SISSO models generated in Eqs. 2-4 of the main manuscript by computing the learning curves, as shown in Fig. S4. These plots show the normalised training and validation scores when we vary the size of the training dataset. Moreover, this approach helps to identify whether our dataset is large enough to obtain reliable results. We generated the learning curves by employing the Scikit-learn library available in Python.[S4] The graphs in Fig. S4 exhibit a similar behaviour for all the proposed model, with a small gap between the training and validation score, which confirms the accuracy of our models. Furthermore, both scores remain unchanged when a training set half the size of the original dataset is considered, meaning that there is no need to increase our dataset to improve the training.

6 Testing SISSO algorithm without specific outliers

A further analysis in creating predicting models for the adhesion of heterogeneous interfaces is to groups the different materials in more homogeneous sets. For example, we can consider only the transition metals and excluding Al, Mg and Ti. We made this choice because Al and Mg are the only simple metals present in our set, whereas Ti requires an exceedingly large number of atomic layers to get a converged surface energy influencing the calculation

of adhesion performed on thinner slabs. When we apply the SISSO algorithm in this new subset, we obtained the following relationships:

$$\mathbf{\Phi}_0 \Longrightarrow \mathrm{E_{adh}} = A_0 + B_0 \cdot \gamma_{GM} + C_0 \cdot \varepsilon_{GM} + \frac{D_0}{A_{GM}} \tag{1}$$

$$R^2_{\mathbf{\Phi}_0} = 0.91 \qquad \mathrm{RMSE}_{\mathbf{\Phi}_0} = 0.39\ \mathrm{J/m^2}$$

$$\begin{aligned} \mathbf{\Phi}_1 \Longrightarrow \mathrm{E_{adh}} = A_1 + B_1 \cdot \gamma_{GM}^2 + C_1 \cdot \frac{\varepsilon_{GM}}{A_{GM}} \\ + D_1 \cdot \gamma_{GM} \cdot (\chi_1 - \chi_2) \end{aligned} \tag{2}$$

$$R^2_{\mathbf{\Phi}_1} = 0.91 \qquad \mathrm{RMSE}_{\mathbf{\Phi}_1} = 0.38\ \mathrm{J/m^2}$$

$$\begin{aligned} \mathbf{\Phi}_2 \Longrightarrow \mathrm{E_{adh}} = A_2 + B_2 \cdot \frac{\gamma_{GM} \cdot \sqrt{\varepsilon_{GM}}}{A_{GM}} + C_2 \cdot (\chi_1 - \chi_2) \cdot \gamma_{GM}^3 \\ + D_2 \cdot \frac{(\chi_1 - \chi_2) \cdot \gamma_{GM}^2}{A_{GM}} \end{aligned} \tag{3}$$

$$R^2_{\mathbf{\Phi}_2} = 0.92 \qquad \mathrm{RMSE}_{\mathbf{\Phi}_2} = 0.36\ \mathrm{J/m^2}$$

Table S3: Coefficients with corresponding units for Eq. 1, 2 and 3.

Model	A_i	B_i	C_i	D_i
$\mathbf{\Phi}_0$	$1.53\ \frac{\mathrm{J}}{\mathrm{m^2}}$	-1.15	$-0.29\ \frac{\mathrm{J}}{\mathrm{m^2\,eV}}$	$8.64\ \frac{\mathrm{J\,Å^2}}{\mathrm{m^2}}$
$\mathbf{\Phi}_1$	$-0.44\ \frac{\mathrm{J}}{\mathrm{m^2}}$	$-0.27\ \frac{\mathrm{m^2}}{\mathrm{J}}$	$-2.41\ \frac{\mathrm{Å^2\,J}}{\mathrm{m^2\,eV}}$	-0.12
$\mathbf{\Phi}_2$	$-0.75\ \frac{\mathrm{J}}{\mathrm{m^2}}$	$-3.92\ \frac{\mathrm{Å^2}}{\sqrt{\mathrm{eV}}}$	$-0.21\ \frac{\mathrm{m^4}}{\mathrm{J^2}}$	$-3.08\ \frac{\mathrm{Å^2\,m^4}}{\mathrm{J^2}}$

These prediction, compared to those present in the main manuscript, are significantly better: the correlation coefficient is above 0.9 for all the feature spaces and the RMSE is reduced between 20% and 24%.

7 Considering additional descriptors for SISSO predictions

As suggested in the main manuscript, we can increase the number of descriptors to further reduce the RMSE. In particular, we identified additional descriptors about the surface com-

mensurability by using the geometric average of the ratios between the slab and supercell areas defined as follows:

$$A^{ratio} = \frac{A_{slab}}{A_{supercell}} \tag{4}$$

We also identified the geometric average of the fractional part in the ratio between the supercell and slab areas as a term of commensurability between the elements present in an interface. We defined this quantity as:

$$FP = \left| \frac{A_{supercell}}{A_{slab}} - \left\lfloor \frac{A_{supercell}}{A_{slab}} \right\rfloor \right| \tag{5}$$

By using these new descriptors, we obtained the following relationships:

$$\mathbf{\Phi}_0 \Longrightarrow \mathrm{E_{adh}} = A_0 + B_0 \cdot \gamma_{GM} + C_0 \cdot \varepsilon_{GM} + \frac{D_0}{A_{GM}} \tag{6}$$

$$R^2_{\mathbf{\Phi}_0} = 0.91 \qquad \mathrm{RMSE}_{\mathbf{\Phi}_0} = 0.39\ \mathrm{J/m^2}$$

$$\mathbf{\Phi}_1 \Longrightarrow \mathrm{E_{adh}} = A_1 + B_1 \cdot \frac{\gamma_{GM}}{A_{GM}} + C_1 \cdot \varepsilon^2_{GM} + D_1 \cdot \gamma_{GM} \cdot FP_{GM} \tag{7}$$

$$R^2_{\mathbf{\Phi}_1} = 0.92 \qquad \mathrm{RMSE}_{\mathbf{\Phi}_1} = 0.37\ \mathrm{J/m^2}$$

$$\mathbf{\Phi}_2 \Longrightarrow \mathrm{E_{adh}} = A_2 + B_2 \cdot \frac{\sqrt{K_{GM}} \cdot \gamma_{GM}}{A_{GM}} + C_2 \cdot \frac{\gamma^3_{GM}}{A^{ratio}_{GM}} + D_2 \cdot \frac{(\chi_1 - \chi_2) \cdot A^{ratio}_{GM} \cdot FP_{GM}}{A_{GM}} \tag{8}$$

$$R^2_{\mathbf{\Phi}_2} = 0.93 \qquad \mathrm{RMSE}_{\mathbf{\Phi}_2} = 0.35\ \mathrm{J/m^2}$$

Table S4: Coefficients with corresponding units for Eq. 6, 7 and 8.

Model	A_i	B_i	C_i	D_i
$\mathbf{\Phi}_0$	$1.53\ \frac{\mathrm{J}}{\mathrm{m^2}}$	-1.15	$-0.29\ \frac{\mathrm{J}}{\mathrm{m^2\ eV}}$	$8.64\ \frac{\mathrm{J\ \mathring{A}^2}}{\mathrm{m^2}}$
$\mathbf{\Phi}_1$	$-0.50\ \frac{\mathrm{J}}{\mathrm{m^2}}$	$-7.29\ \mathrm{\mathring{A}^2}$	$-0.033\ \frac{\mathrm{J}}{\mathrm{m^2\ eV^2}}$	0.67
$\mathbf{\Phi}_2$	$-0.68\ \frac{\mathrm{J}}{\mathrm{m^2}}$	$-0.67\ \frac{\mathrm{\mathring{A}^2}}{\sqrt{\mathrm{GPa}}}$	$0.0019\ \frac{\mathrm{m^4}}{\mathrm{J^2}}$	$206.34\ \mathrm{\mathring{A}^2}$

Comparing these formulas with the ones shown in the previous section, we find a slight

increase of 0.01 in the correlation coefficient for all the relationships and a reduction of the RMSE of about 3%.

References

(S1) Kittel, C. *Introduction to Solid State Physics*, 8th ed.; John Wiley & Sons, 2004.

(S2) Jain, A.; Ong, S. P.; Hautier, G.; Chen, W.; Richards, W. D.; Dacek, S.; Cholia, S.; Gunter, D.; Skinner, D.; Ceder, G.; Persson, K. A. Commentary: The Materials Project: a Materials Genome Approach to Accelerating Materials Innovation. *APL Materials* **2013**, *1*, 011002.

(S3) Wolloch, M.; Losi, G.; Chehaimi, O.; Yalcin, F.; Ferrario, M.; Righi, M. C. High-Throughput Generation of Potential Energy Surfaces for Solid Interfaces. *Computational Materials Science* **2022**, *207*, 111302.

(S4) Pedregosa, F.; Varoquaux, G.; Gramfort, A.; Michel, V.; Thirion, B.; Grisel, O.; Blondel, M.; Prettenhofer, P.; Weiss, R.; Dubourg, V.; Vanderplas, J.; Passos, A.; Cournapeau, D.; Brucher, M.; Perrot, M.; Duchesnay, E. Scikit-learn: Machine Learning in Python. *Journal of Machine Learning Research* **2011**, *12*, 2825–2830.
